# Effect of *In Vitro* Digestion on the Phenolic Content of Herbs Collected from Eastern Anatolia

**DOI:** 10.1021/acsomega.2c07881

**Published:** 2023-03-28

**Authors:** Gulay Ozkan, Fatma Betul Sakarya, Dilara Tas, Bayram Yurt, Sezai Ercisli, Esra Capanoglu

**Affiliations:** † Department of Food Engineering, Faculty of Chemical and Metallurgical Engineering, Istanbul Technical University, 34469 Maslak, Istanbul, Turkey; ‡ Department of Food Engineering, Faculty of Engineering and Architecture, Bingol University, Bingol 12300, Turkey; § Department of Horticulture, Faculty of Agriculture, Ataturk University 25240, Erzurum, Turkey

## Abstract

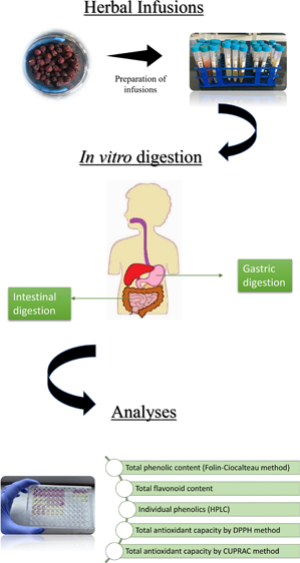

Phenolic compounds in herbs have high antioxidant activities, and their consumption as functional foods may impact human health positively. The main objective of this study was to investigate the total phenolic (TPC) and total flavonoid (TFC) contents as well as total antioxidant capacities (TAC) of bioactive compounds in various infusions prepared by herbs collected from the Bingöl region of Turkey during *in vitro* gastrointestinal digestion. According to the results, while the highest TPC (5836 ± 373 mg GAE/100 g dw), TFC (2301 ± 158 CE/100 g, dw), and TAC (1347 mg TE/100 g dw) were obtained with *Anchusa azurea* Mill. species, *Crataegus orientalis* exhibited the lowest values (863 ± 24 mg GAE/100 g dw, 242 ± 23 CE/100 g dw, 735 ± 47 mg TE/100 g dw, respectively). Gallic acid and chlorogenic acid were the most common phenolic compounds in the infusions. In detail, the highest gallic acid was found in *E. spectabilis* M. Bieb (27.3 ± 0.9 mg/100 g of dw) and the highest chlorogenic acid was observed in *F. elaeochytris* (919.2 ± 35.7 mg/100 g of dw). After *in vitro* gastrointestinal digestion, the highest bioaccessibility values of TPC and TFC were determined as 0.6- and 3-fold of the values observed in undigested *C. orientalis*, respectively. Besides, *C. orientalis* Pall. had the highest bioaccessibility of TAC according to the DPPH (6.7-fold increase) and CUPRAC (9.7-fold increase) assays. It can be concluded that the use of these medicinal herbs in human dietary intake due to their high bioactive compounds even after digestion can improve nutritional value and contribute to human health.

## 1 Introduction

Antioxidants are defined as part of the defense mechanism of higher organisms and prevent diseases related to oxidative stress that contribute to the production of free radicals and reactive metabolites by reducing the amount of free radical formation, preventing its negative consequences, and supporting the body’s natural antioxidant and detoxification systems. According to the literature, herbs are good sources of bioactive compounds including mainly phenolics and flavonoids with significant antioxidant activity, which have potential health benefits.^1−3^ Phenolic compounds are thought to be complementary compounds in the treatment of several diseases, including mental disorders, with their antioxidant, anti-inflammatory, and neuroprotective properties.^4^ For example, clinical evidence has confirmed that the neuroinflammatory and apoptotic pathway is reduced by a natural phenolic component, hesperidin.^5^ Therefore, infusions/teas made from these plants are of great interest due to their high phenolic contents.^6,7^

Most of the world population uses herbal products for primary health care, and these products are preferred due to their effects on preventing common diseases with natural bioactive compounds.^8,9^ Leahu et al.^10^ mentioned that rosehip, which includes a significant amount of vitamin C, has long been used in the treatment and traditional prevention of the common cold and other inflammatory diseases. The herbs investigated in this study, *Rosa canina* L., *Crataegus monogyna* Jacq., *Crataegus orientalis* Pall., *Crataegus orientalis*, *Iris persica* L., *Ferula elaeochytris*, *Anchusa azurea* Mill., and *Eremurus spectabilis* M. Bieb., are known to contain high amounts of phenolics with antioxidant activity. While some of these herbs (*R. canina, C. orientalis*) are common worldwide, some of them are endemic in the east region of Turkey. These herbs have been usually consumed as herbal tea (*Rosa canina* L), jam (*Rosa canina* L., *Crataegus monogyna* Jacq., *Crataegus orientalis* Pall., *Crataegus orientalis*), or juice (*Crataegus monogyna* Jacq., *Crataegus orientalis* Pall., *Crataegus orientalis*) or by cooking (*Anchusa azurea* Mill., *Eremurus spectabilis* M. Bieb). It is worth noting that the antioxidant capacity was determined using infusions, which is the most common way for these herbs to be consumed by the general population. However, there is limited data available in the literature, with the majority of results focusing on hydroalcoholic extracts of these herbs.

Estimating the bioaccessibility of consumed polyphenols, which can be defined as the number of bioactive components released during gastrointestinal digestion and made available for intestinal absorption, is the first step in understanding the digestive fate of polyphenols. Polyphenols may interact with other constituents of food, be metabolized, or be degraded during gastrointestinal digestion.^7^ The pharmacological activity of antioxidant molecules is expected to be induced by their absorption through the intestinal membrane. In order to determine the bioaccessibility of bioactive compounds, *in vitro* gastrointestinal digestion simulation models have been widely conducted.^12^

Considering the above, this study aimed to determine and compare the antioxidant capacity of 8 different herbal infusions during *in vitro* digestion and shed light on future studies related with these plants. For this purpose, herbal infusions were prepared, and phenolic compounds and antioxidant capacities with two common methods (CUPRAC and DPPH) were assessed. After spectrophotometric analyses, chromatographic analyses were performed with HPLC-PDA to determine the profile of phenolic compounds in plant infusions before and after *in vitro* digestion. Later, changes in the content and antioxidant capacity of bioactive compounds in herbal infusions after gastrointestinal digestion were investigated.

## 2 Materials and Methods

### 2.1 Materials

Some of the plants (*Iris persica* L., *Ferula elaeochytris*, *Anchusa azurea* Mill., *Eremurus spectabilis* M. Bieb) were collected in the spring season (April, May 2022); others (*Rosa canina* L., *Crataegus monogyna* Jacq., *Crataegus orientalis* Pall., *Crataegus orientalis*) were collected in the fall season (September, October 2022). Samples of rosehip, hawthorn, oriental hawthorn, and ferula were provided as a dried form, whereas other samples were provided as a fresh form and freeze-dried before infusion preparation.

The analytical or HPLC-grade chemicals, standards, and enzymes (pepsin (541 U/mg, EC 3.4.23.1), pancreatin (8× USP, EC 232.468.9)) used in the analyses were all purchased from Sigma-Aldrich (Steinheim, Germany). Herbs used in this work were obtained as three independent biological replicates from the Eastern Anatolia Region, Turkey (Table 1).

**Table 1:** Scientific and Common Names of Herbs Used in This Study

Plant Name (Latin)	Common Names
*R. canina* L.	Rosehip
*C. monogyna* Jacq.	Hawthorn
*C. orientalis* Pall.	Oriental Hawthorn
*C. orientalis*	Oriental Hawthorn
*I. persica L.*	Persian Iris
*F. elaeochytris*	Ferula
*A. azurea* Mill.	Garden Anchusa, Loddon Royalist
*E. spectabilis* M. Bieb.	Foxtail Lilly

### 2.2 Infusion Preparation

All samples were ground for 1 min in a coffee grinder (Sinbo, SCM 2934) and stored at room temperature until use. The treatments were divided into three independent groups (*n* = 3). Infusion preparation was adapted from previous procedures.^7,13^ The sample amount was fixed at 10% (w/v) in all infusions. A certain amount of sample was weighed into a beaker, 85 °C water was added, and the mixture was left for 10 min without heating. The mixture was then cooled and filtered through Whatman No. 4 paper. All infusions were kept at −20 °C until further testing.

### 2.3 Extraction of Polyphenols

Extractions were performed according to the procedure previously described in the study of Capanoglu et al.^14^ After weighing 1.00 g of ground samples into 50 mL of falcon tubes, 5 mL of 75% methanol solution was added. The prepared mixtures were vortexed for 10 s and kept in an ultrasonic bath (USC900TH; VWR, Radnor, PA, USA) for 15 min. All samples were centrifuged at 2700*g* (4000 rpm) for 10 min at 4 °C (Universal 32R; HettichZentrifugen, Tuttlingen, Germany), and the supernatants were collected. These processes were repeated once again, and the two upper phases were pooled and completed to a volume of 10 mL. The prepared extracts were stored at −20 °C for use in subsequent analyses.

### 2.4 *In Vitro* Simulated Gastrointestinal Digestion

The *in vitro* gastrointestinal digestion protocol was conducted on the basis of the method reported by Minekus et al.,^15^ with minor modifications. This method involves simulating buccal, gastric, and intestinal digestion steps in sequence.

To simulate oral digestion, 5 mL of each herbal infusion was combined with 4 mL of salivary juice, 25 μL of 0.3 mol/L CaCl_2_, and 975 μL of distilled water. For 2 min, this mixture was incubated at 37 °C in a shaking water bath (Memmert SV 1422, Memmert GmbH & Co. Nürnberg, Germany).

Gastric digestion was simulated by adding 7.5 mL of gastric juice, 1.6 mL of pepsin solution (417 μkat/mL), and 5 μL of 0.3 mol/L CaCl_2_ to the remaining oral bolus, and the pH was adjusted to 3.0 with 0.2 mL of 1 mol/L HCl. Using distilled water, the total volume of this mixture was reduced to 20 mL. The mixture was then incubated for 2 h in a shaking water bath at 37 °C. After simulated gastric digestion, 5 mL aliquots of each infusion were separated for gastric digestion analysis.

The gastric chyme was mixed with 8.25 mL of intestinal juice, 3.75 mL of pancreatin (13 μkat/mL), 1.875 mL of 160 mmol/L bile, and 30 μL of 0.3 mol/L CaCl_2_. Using 1 mol/L NaOH, the pH of the mixture was adjusted to 7.0. Using distilled water, the total volume of this mixture was completed to 30 mL. For 2 h, the mixture was incubated in a shaking water bath at 37 °C.

In order to correct for any interference from the simulated digestive fluids, a blank (the same amount of water used instead of samples) was also incubated under the same conditions. All samples from the simulated gastric and intestinal digestion steps were centrifuged for 5 min at 23000*g* and 4 °C (Hettich, Tuttlingen, Germany). The supernatants were stored at −20 °C until further analysis.

### 2.5 Identification and Quantification of Polyphenols by HPLC-PDA

The method of Capanoglu et al.^14^ was used to quantify polyphenols in the samples. Concisely, samples were passed through 0.45 μm membrane filters before being injected into a Waters 2695 HPLC system with a PDA detector. The stationary phase was a A Supelcosil LC-18 (25 cm × 4.60 mm, 5 m column, Sigma-Aldrich, Steinheim, Germany).

TFA/MQ water (1 mL/L; eluent A) and TFA/acetonitrile (1 mL/L; eluent B) were the solvents used for the spectral measurements at λ = 280, 312, and 360 nm and had flow rates of 1 mL/min and injection volumes of 10 mL, respectively.

A linear gradient was used as follows: At 0 min, 95% solvent A and 5% solvent B; at 45 min, 65% solvent A and 35% solvent B; at 47 min, 25% solvent A and 75% solvent B; and at 54 min, returning to initial conditions. Phenolic acids were quantified by using their authentic standards. All analyses were carried out in triplicate, and the results were expressed as mg/100 g of dry weight of the sample.

### 2.6 Spectrophotometric Analyses

TPC was determined using the Folin–Ciocalteau method specified in the study of Singleton and Rossi.^16^ A 15 μL portion of extract and 112.5 μL of Folin–Ciocalteu reagent were mixed and incubated at room temperature for 5 min. Then, 112.5 μL of 6% Na_2_CO_3_ solution was added to the mixture and incubated for 60 min at room temperature and in a dark environment. After the incubation period, absorbance values were measured at λ = 765 nm using a spectrophotometer. The calibration curve was used to determine the total phenolic content of the analyzed samples (*R*^2^ = 0.9992, *y* = 3.6822*x* – 0.0147), and the results were expressed as milligrams of gallic acid equivalents (GAE)/100 g of dry weight.

TFC was determined using a method applied by Dewanto et al.^17^ The principle is based on the adhesion of Al to the cyclic structure and causes a color change with the effect of NaOH. 75 μL of 5% NaNO_2_ was added to 0.25 mL of extract and left at room temperature for 6 min. Then, 150 μL of 10% AlCl_3_·H_2_O was added, and after waiting for 5 min, 500 μL of 1 M NaOH was added to the mixture. The volume was adjusted to 2.5 mL by adding 1525 μL of distilled water, and absorbance was measured at λ = 510 nm with a spectrophotometer. The *T* curve was used to determine the total flavonoid substance amounts of the analyzed samples (*R*^2^ = 0.993, *y* = 2.15*x* – 0.0318), and the results were obtained in mg catechin equivalents (CE) /100 g dry weight.

In this study, two different methods were applied for antioxidant capacity analysis. TAC was determined by the DPPH (1,1-diphenyl-2-picrylhydrazil) method specified by Brand-Williams et al.^18^ A 10 μL portion of extract and 200 μL of DPPH reagent dissolved in 0.1 mM methanol were mixed and left to incubate for 30 min in the dark environment and at room temperature after shaking for 10 s. At the end of the incubation, absorbance was measured at λ = 517 nm. The calibration curve was used to determine the total antioxidant capacity of the analyzed samples (*R*^2^ = 0.990, *y* = 4.0161*x* + 0.0792), and the results were expressed as mg Trolox equivalents (TE)/100 g dry weight.

The copper(II) ion reducing antioxidant capacity (CUPRAC) method was performed using the method of Apak et al. A 70 μL portion of 10 mM copper(II) chloride, 70 μL of 7.5 mM neocuproine, 70 μL of 1 mM ammonium acetate (pH: 7.0), and 70 μL of distilled water were added to 7 μL of extract, after shaking for 10 s at room temperature, and incubated for 30 min in the dark. When the incubation period was over, absorbance was measured at λ = 450 nm. The total antioxidant capacity of the analyzed samples was determined using the calibration curve (*R*^2^ = 0.991, *y* = 2.4117*x* – 0.0164), and the results were expressed as mg Trolox equivalents (TE)/100 g dry weight.

### 2.7 Statistical Analysis

Upon confirmation that all data exhibit normality, all analyses were made by performing three parallel measurements from the samples prepared in three replications. The expression of the obtained results is in the form of the mean ± standard deviation, and the data were evaluated by one-way analysis of variance (ANOVA) in SPSS (28.00). Differences between samples were determined by the Scheffe test (*p* < 0.05). The spectrophotometric analyses’ correlation coefficients (*R*_2_) were calculated by using Excel (Microsoft, USA) software.

## 3 Results and Discussion

### 3.1 Phenolic Compounds of Herbal Extracts and Their Antioxidant Capacities

**Figure 1:**
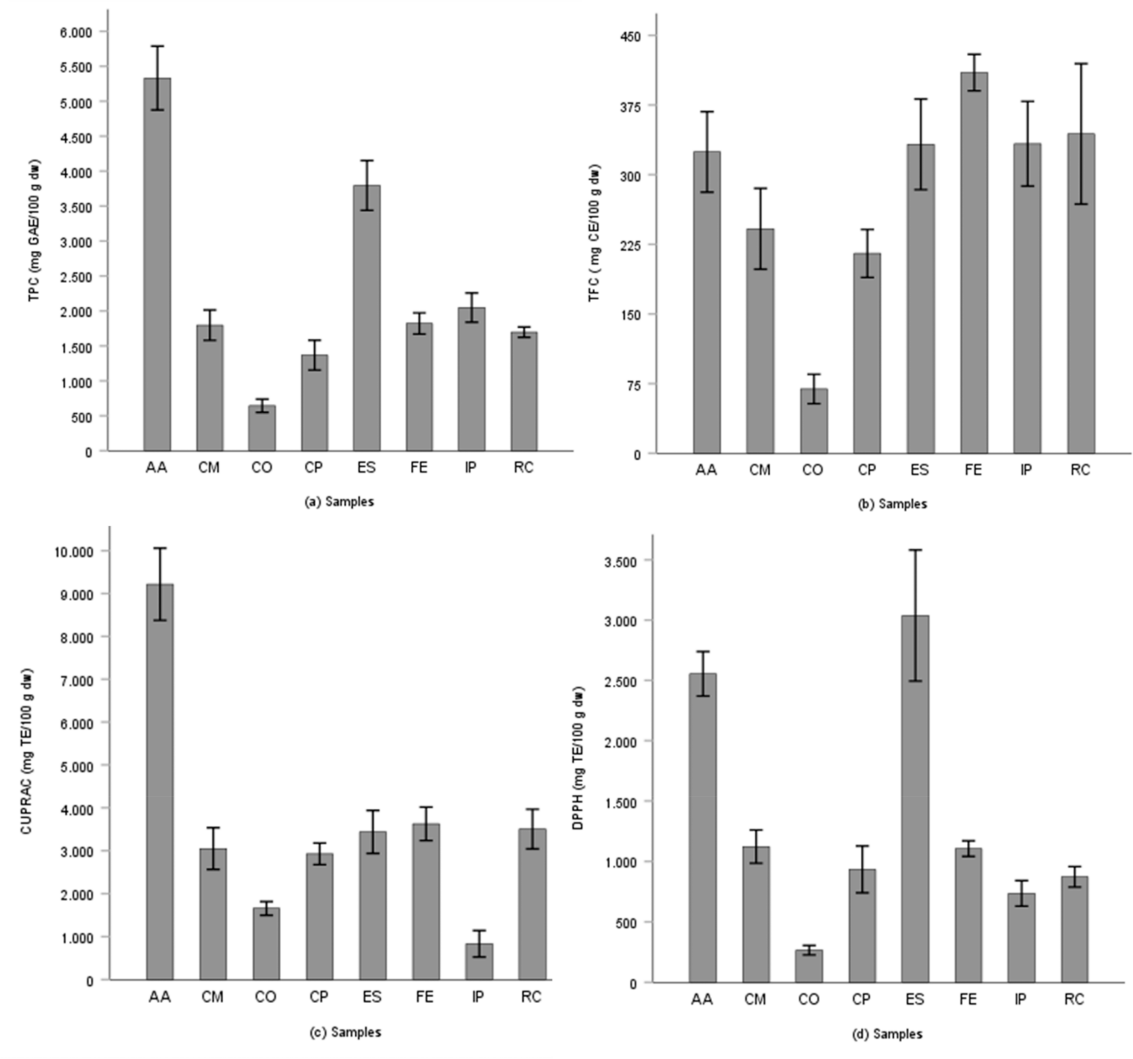
Effect of *in vitro* digestion on phenolic compounds of herbal infusions: (a) TPC: total phenolic content by gallic acid equivalents. (b) TFC: total flavonoid content by catechin equivalents. (c) CUPRAC: cupric ion reducing antioxidant capacity. (d) DPPH: 1,1-diphenyl-2-picrylhydrazyl by Trolox equivalents. AA, *A. azurea*; CM, *C. monogyna Jacq.*; CO, *C. orientalis*; CP, *C. orientalis Pall.*; ES, *E. spectabilis*; FE, *F. elaeochytris*; IP, *I. persica*; RC, *R. canina*.

The total phenolic and total flavonoid contents of the methanolic extracts of the samples are listed in Figure 1. According to the results, there were statistically significant differences between the total phenolic contents of the samples (*p* < 0.05). The highest total phenolic content was found to be 5331 ± 457 mg GAE/100 g dw in *A. azurea* Mill, followed by *E. spectabilis* M. Bieb (3795 ± 356 mg GAE/100 g dw) and *I. persic*a L. (2048 mg of GAE/100 g of dw) samples. Besides, it was determined that the total phenolic content values of *F. elaeochytris* (1820 ± 152 mg GAE/100 g dw), *C. monogyna* Jacq. (1796 ± 217 mg GAE/100 g dw), and *R. canina* L. (1696 ± 73 mg of GAE/100 g of dw) were close to each other.

The total phenolic content of the *A. azurea* root extract was found as 4329 ± 112 mg GAE/100 g dw in the literature,^19^ which was similar to the values obtained in this study. Moreover, it was determined that the total phenolic content of the *R. canina* L. sample was close to the results of previous studies (1920 ± 1.71 mg GAE/100 g dw).^9^ In contrast, the total phenolic content of *A. azurea* was found to be 302.1 ± 22.1 mg GAE/100 g dw and 232.7 ± 32.6 mg GAE/100 g dw in the samples collected from two different cities of Turkey, which were much lower than the values found in the present study.^20^ In a study conducted by Javid et al.,^21^ the total phenolic content (6161 ± 1.3 mg GAE/100 g dw) detected in the *R. canina* L. sample collected from Denizli-Turkey province was much higher than the value provided in this study (1696 ± 73 mg GAE/100 g dw). The total phenolic content in the *E. spectabilis* M. Bieb sample collected from the province of Bingol-Turkey, which had the highest value after the garden anchusa plant in this study, was higher than the values found in the samples obtained by Murathan et al.^2^ from the same province (115.8 ± 10.2 mg of GAE/100 g of dw). These differences can be attributed to the region where the plant grows, meteorological and petrographic changes, the harvest season, and the vegetative stage of the plant.^19^ Moreover, this inconsistency between values of the present and the aforementioned study may also arise from the type of extraction method, type of solvent, and solvent-to-solid ratio.^2^

### 3.2 Retention of Phenolics during *In Vitro* Gastrointestinal Digestion

Because of their chemical instability in the gastrointestinal tract and relatively poor water solubility, there is a limit on the use of polyphenols. Therefore,explaining the metabolism and absorption mechanism as well as their bioactivity in the target tissue is essential to investigate the recovery and durability of the polyphenols throughout digestion.^22^Table 2 demonstrates the changes in the phenolic compounds of herbal infusions during *in vitro* digestion.

**Table 2:** Effect of *In Vitro* Digestion on Phenolic Compounds of Herbal Infusions^a^

		Treatments			
Assays	Infusions	UD	GD	ID	% Bioaccessibility
TPC (mg GAE/100 g)	*R. canina* L.	3765 ± 350 ^**bA**^	2636 ± 368 ^**bB**^	1860 ± 165 ^**bC**^	49 ± 4^bc^
	*C. monogyna* Jacq.	1757 ± 199^deA^	1433 ± 156 ^deB^	278 ± 34 ^cC^	16 ± 2^e^
	*C. orientalis* Pall.	1257 ± 161^efA^	883 ± 66 ^efB^	500 ± 68 ^cC^	40 ± 5^cd^
	*C. orientalis*	863 ± 24 ^fA^	701 ± 81 ^fB^	524 ± 55 ^cC^	61 ± 6^a^
	*I. persica* L.	2878 ± 119 ^cB^	5294 ± 571^aA^	1687 ± 210 ^**bC**^	59 ± 7^ab^
	*F. elaeochytris*	2952 ± 391 ^cA^	1755 ± 217 ^cdB^	1602 ± 253 ^**bB**^	54 ± 9^ab^
	*A. azurea* Mill.	5836 ± 373 ^aA^	5096 ± 280 ^aB^	3139 ± 394 ^aC^	54 ± 7^ab^
	*E. spectabilis* M. Bieb.	1835 ± 227 ^dB^	2175 ± 278 ^**bcA**^	690 ± 69 ^cC^	38 ± 4^d^
TFC (mg CE/100 g)	*R. canina* L.	656 ± 76 ^cB^	1229 ± 137 ^**bA**^	793 ± 37 ^dB^	121 ± 6^e^
	*C. monogyna* Jacq.	562 ± 49 ^cdB^	416 ± 51 ^cdC^	792 ± 20 ^dA^	141 ± 4^d^
	*C. orientalis* Pall.	416 ± 59 ^deB^	298 ± 42 ^dC^	675 ± 75 ^deA^	162 ± 18^c^
	*C. orientalis*	242 ± 23 ^eB^	271 ± 25 ^dB^	744 ± 38 ^dA^	307 ± 16^a^
	*I. persica* L.	743 ± 24^cC^	1002 ± 98 ^bB^	1327 ± 53 ^bA^	179 ± 7^b^
	*F. elaeochytris*	1337 ± 148^bA^	1186 ± 249 ^**bA**^	1116 ± 104 ^cA^	83 ± 8^f^
	*A. azurea* Mill.	2301 ± 158^aC^	3746 ± 156 ^aA^	2723 ± 145 ^aB^	118 ± 6^e^
	*E. spectabilis* M. Bieb.	598 ± 31^cA^	630 ± 63 ^cA^	435 ± 13 ^deB^	73 ± 2^f^

a UD: undigested digestion, GD: gastric digestion, ID: intestinal digestion; TPC: total phenolic content; TFC: total flavonoid content; GAE: gallic acid equivalents; CE: catechin equivalents. The data presented in this table consist of average values ± standard deviation of three independent batches. Different small letters indicate the differences between different herb species, while capital letters represent the statistically significant differences between the digestive phases of the same herb sample (*p* < 0.05).

TPC and TFC values were found to be significantly different (*p* < 0.05) for samples of undigested, *in vitro* gastric digested, and *in vitro* intestinal digested. Between the undigested samples, *A. azurea* showed the highest TPC with 5836 ± 373 mg GAE/100g dw, which was almost 65% greater amount than *R. canina,* which had the second highest TPC (3765 ± 350 mg GAE/100 g dw). On the other hand, according to Petkova et al.,^23^ the TPC of *R. canina* was found as 2680 ± 10 mg GAE/100 g dw. Differences in the content and phenolic compound profile in herbal infusions between research studies may arise from various factors like plant growth area, the amount of plant sample used for the preparation of infusion, harvest processing, etc., as reported in previous studies.^23,24^ In addition to these, the lowest values were obtained for *C. orientalis* and *C. orientalis* Pall. as 863 ± 24 and 1257 ± 161 mg GAE/100 g dw, respectively. The data obtained for *C. orientalis* species in this study complies with a previous study on phenolics of hawthorn (*Crataegus* spp.) obtained from Poland, with the TPC values in the range of 16–1207 mg GAE/100.^25^

Estimating the bioaccessibility of polyphenols is essential for understanding their digestive fate, as they may interact with other constituents, be metabolized, or be degraded during digestion.^7,11^ Considering our results, a decrease in TPC values during gastrointestinal digestion was obtained except for the samples *I. persica* and *E. spectabilis*, which showed an increase in the phenolic contents for postgastric digestion. Furthermore, even the undigested sample of *I. persica* had a lower TPC compared to the *A. azurea*; after gastric digestion, these samples showed similar and the highest TPC results. As Qin et al.^26^ mentioned, it was anticipated that some phenolic compounds that are bound to carbohydrates would be released by pepsin action and low pH during gastric digestion, making these bioactive chemicals more bioaccessible. The bioaccessibility of phenolic compounds in all hawthorn species showed a decrease during the simulated gastric digestion. Similar to the results obtained in the present work, Lou et al.^27^ found that the TPC of *C. pinnatifida* fruit (hawthorn berry) after gastric digestion decreased significantly. On the other hand, even when the infusion prepared by *A. azurea* was observed to be rich in polyphenols, TPC bioaccessibility during intestinal digestion of *C. orientalis* was higher (61 ± 6%) than the rest of the samples, followed by *I. Persica* (59 ± 7%), *F. elaeochytris* (54 ± 9%), and *A. azurea* (54 ± 7%). Besides, *A. azurea* showed the highest result of TPC with 3139 ± 394 mg GAE/100 g dw. After simulated intestinal digestion, the bioaccessibility of the TPC of *R. canina* was calculated as 49.4% (almost 50% loss in phenolic content). These results are in agreement with the findings of Celep et al.,^12^ who reported an approximately 57% decrease in TPC during intestinal digestion. TPC results showed both an increase and a decrease during gastrointestinal digestion. Other studies on various food samples reporting a rise in the TPC after *in vitro* gastric digestion and demonstrating a significant reduction in the total phenolics postdigestion were in agreement with our results, which indicates that the effect of this in vitro simulation process differs depending on the bioactive compound in foods, its nature, and its concentration.^28,29^ It has been reported that loss in the phenolics was due to autoxidation, polymerization, and epimerization under the digestion conditions. In fact, phenolic substances may degrade throughout gastrointestinal digestion because of their low alkaline stability.^30^

Similar to TPC results of undigested samples, *A. azurea* showed the highest TFC value (2301 ± 158 mg CE/100 dw) followed by *F. elaeochytris* (1337 ± 148 mg CE/100 dw), *I. Persica* (743 ± 24 mg CE/100 dw), and *R. canina* (656 ± 76 mg CE/100 dw). Although the TPC value was obtained much higher for *R. canina* than for *F. elaeochytris*, the TFC result of *R. canina* was determined to be significantly lower, which may imply that the phenolic acids in rosehip were more dominant than the flavonoids. In the study performed by Polumackanycz et al.^31^ using 20 different *R. canina* samples, the TFC results of infusions concurred with our findings. On the other hand, TFC of undigested *R. canina* as well as *C. monogyna* and *C. orientalis* were found to be 3983 ± 84 mg QE/100 g dw (QE; quercetin equivalent)^12^ and 577 ± 9 mg QE/100 g dw, respectively.^32^

After simulated gastric digestion, an increase in the TFC was observed for most of the samples, in contrast to the TPC results. Considering the findings of the present study, although *A. azurea* had the highest TFC with an increase of 163% after GD, the greater bioaccessibility in TFC was determined for *R. canina*, with a value of 187%. Qin et al.^26^ indicated that the amounts of total flavonoids were significantly higher for raspberry species after simulated gastric digestion. Moreover, in a previous study, an increase in TFC of hawthorn infusions after simulated gastric digestion was indicated, in parallel to our findings. On the other hand, even similar TFC postgastric results for *R. canina* were obtained by Celep et al.,^12^ and approximately 54% reduction in TFC of *R. canina* was reported contrary to our work. Similar to the results for TFC after GD, the results of the present study demonstrated an increase in intestinal-digested TFC. Surprisingly, the highest bioaccessibility of TFC after ID (307 ± 6%) was observed for *C. orientalis*, while the undigested TFC of this sample showed the lowest results. On the other hand, the lowest bioaccessibilities of TFC after ID were obtained for *E. spectabilis* and *F. elaeochytris* as 73 ± 2–83 ± 8%, respectively, followed by *A. azurea* (118 ± 6%) and *R. canina* (121 ± 6%). In a study conducted by Ozkan et al.,^33^ the bioaccessibility of TFC in rosehip samples was found to range from 23.4% to 30.4%, which was significantly lower than the result of the present study, whereas Celep et al.^12^ reported a slightly lower value for rosehip with 49% bioaccessibility. Qin et al.^26^ reported that the highest content of total flavonoid for raspberry samples was obtained for intestinal digestion and mentioned that various phenolic compounds might be released during the ID. This can be explained by some phenolic contents that are bound to proteins tend to be hydrolyzed and released by the impact of digestive enzymes and different pH conditions, and therefore, the extraction of them can be more efficient. Moreover, based on the basis of their chemical structure, polyphenols are affected by the action of intestinal enzymes differently, contributing to derivatives that may have a strong effect on their antioxidant activity.^34^

In this study, phenolic substances obtained in infusions were estimated by both chromatographic and spectrophotometric analysis because reagents or chemicals used in spectrophotometric analyses may interact with food components (reducing sugars, some amino acids, reducing compounds such as citric acid and dehydroascorbic acid), leading to the overestimation of polyphenols and low specificity of the analysis method.^35,36^

Phenolic compounds, including gallic acid, chlorogenic acid, rutin, protocatechuic acid, syringic acid, and quercetin, were quantified in the infusion samples (Table 3) of *E. spectabilis* M. Bieb. The infusion had the highest gallic acid value (27.3 ± 0.9 mg/100g), while *C. monogyna* Jacq. infusion was found to have the highest amount of epicatechin (133.1 ± 10.8 mg/100 g dw). Chlorogenic acid and epicatechin were detected as the common ones in the infusions of all *Crataegus* species (*C. monogyna* Jacq., *C. orientalis* Pall, and *C. orientalis*) analyzed in this study. Apart from these components, protocatechuic acid, gallic acid, rutin, and 2,3,4-trihydroxybenzoic acid were also determined in the *Crataegus* species. Similarly, Zheng et al.^37^ detected chlorogenic acid and epicatechin in the infusion of *C. pinnatifida*, which is from *Crataegus* species. Moreover, although there was a difference in sample preparation technique, gallic acid, protocatechuic acid, and rutin were also indicated in the previous study for hawthorn, which was in agreement with our findings.^27^ On the other hand, while gallic acid was found in *R. canina* primarily followed by protocatechuic acid, catechin was reported as a major phenolic compound followed by ellagic acid, quercetin, chlorogenic acid, and cinnamic acid in the previous study, which was inconsistent with our results.^33^ In that previous study, UPLC-qTOF-MS/MS method was used for the detection of polyphenols; thus, these various results might be caused by the difference in identification method.

**Table 3:** Effect of Simulated *In Vitro* Digestion on Individual Phenolic Compounds of Herbal Infusions^a^

Sample	Component	UD (mg/100 g dw)	GD (mg/100 g dw)	ID (mg/100 g dw)
*R. canina* L.	Gallic acid	18.0 ± 2.3	8.7 ± 3.2	100.7 ± 1.8
	Protocatechuic acid	2.9 ± 0.5	n.d.	n.d.
*C. monogyna* Jacq.	2,3,4-trihydroxy benzoic acid	2.0 ± 0.3	n.d.	n.d.
	Epicatechin	133.1 ± 10.8	63.8 ± 0.4	n.d.
	Chlorogenic acid	10.1 ± 1.5	n.d.	n.d.
	Rutin	10.2 ± 0.9	11.6 ± 1.7	n.d.
*C. orientalis* Pall.	Protocatechuic acid	1.9 ± 0.1	n.d.	n.d.
	Epicatechin	45.1 ± 2.4	n.d.	n.d.
	Epigallocatechin	8.7 ± 0.9	n.d.	n.d.
	Chlorogenic acid	6.4 ± 0.5	11.6 ± 2.9	n.d.
	Rutin	29.7 ± 4.2	9.6 ± 0.03	n.d.
*C. orientalis*	Protocatechuic acid	15.8 ± 2.8	10.9 ± 3.6	43.9 ± 1.4
	Gallic acid	0.5 ± 0.1	3.6 ± 1.7	43.9 ± 1.4
	Epicatechin	3.4 ± 0.3	43.3 ± 6	314.6 ± 13.9
	Chlorogenic acid	2.8 ± 0.1	n.d.	n.d.
*I. persica* L.	Epicatechin	12.7 ± 1.4	n.d.	3360 ± 208
	Epigallocatechin	17.2 ± 0.7	n.d.	n.d.
	Gallic acid	24.7 ± 1.6	17 ± 3.3	119.5 ± 8.6
	Syringic acid	41.4 ± 2.9	42.4 ± 5.9	n.d.
	Chlorogenic acid	465.6 ± 10.4	20.5 ± 1.9	767.6 ± 43.7
	Quercetin	326.4 ± 12	158.6 ± 39.1	n.d.
	Rutin	155.4 ± 5,3	122.9 ± 19.4	693.4 ± 58
*F. elaeochytris*	Gallic acid	5.5 ± 1.4	1.1 ± 0.3	9.6 ± 3.1
	Protocatechuic acid	5.6 ± 0.9	n.d.	n.d.
	Chlorogenic acid	919.2 ± 35.7	814.4 ± 52.4	n.d.
*A. azurea* Mill.	Gallic acid	17.3 ± 5	21.3 ± 2	64.9 ± 3.8
	*trans*-Cinnamic acid	9.8 ± 1.2	n.d.	n.d.
	4-Hydroxybenzoic acid	16.1 ± 2.3	12.3 ± 5.1	n.d.
	Syringic acid	53.5 ± 2.9	39.5 ± 4.2	n.d.
	Chlorogenic acid	46.3 ± 14	n.d.	n.d.
	*p*-Coumaric acid	5 ± 0.05	n.d.	n.d.
	Rutin	103.3 ± 9,7	n.d.	n.d.
*E. spectabilis* M. Bieb.	Gallic acid	27,3 ± 0.9	25.4 ± 5.8	98.8 ± 9.1
	4-Hydroxybenzoicacid	281 ± 19.6	434.6 ± 64.8	n.d.
	Syringic acid	42.6 ± 1.3	34.3 ± 2.8	141.7 ± 21.6
	*p*-Coumaric acid	2.7 ± 0.1	n.d.	n.d.
	Rutin	11.2 ± 4.4	n.d.	134.2 ± 25
	Quercetin	489.4 ± 39.3	384 ± 36.4	n.d.

a Data are expressed as mean ± standard deviation. n.d.: not determined. UD: undigested digestion; GD: gastric digestion; ID: intestinal digestion.

The concentration of most phenolic components (except for rutin, chlorogenic acid, epicatechin, syringic acid, and gallic acid) was decreased by 11.41–95.59% after in vitro gastric digestion in comparison to the results before digestion. The quercetin component was only available in *I. persica L.* (326.4 ± 12) and *E. spectabilis* M. Bieb (489.4 ± 39.3) samples, and a decrease in quercetin content (by 51.4–21.4%, respectively) was observed during the gastric digestion, whereas it was not detected in both samples after the intestinal digestion. The 2,3,4-trihydroxybenzoic acid component was observed only in *C. monogyna* Jacq. infusion and was not detected during *in vitro* gastric digestion. The results of previous research indicated that polyphenols decrease dramatically after *in vitro* digestion. It was indicated that the decrease in the recovery of phenolics could be related to pH changes and the presence of bile salts, which may cause precipitation during the digestion simulation.^30^

Considering the data in Table 3, a significant increase in the content of gallic acid, protocatechuic acid, epicatechin, chlorogenic acid, rutin, and syringic acid was obtained for most of the samples (except for *C. monogyna* Jacq. and *C. orientalis* Pall.) after *in vitro* intestinal digestion. However, phenolics were not detected in *C. monogyna* Jacq. or *C. orientalis* Pall in the intestinal phase. As shown in Table 3, the highest increase was determined for epicatechin in *I. persica* L. (increased from 12.7 ± 1.4 to 3360 ± 208 mg/100 g dw). In addition, it was observed that epicatechin in *C. orientalis* also presented an increase during *in vitro* gastrointestinal digestion, which was in line with the previous study.^37^ Gallic acid was released during *in vitro* intestinal digestion and showed an increase in all samples. The increase in gallic acid content may be directly related to the hydrolysis of gallotannins present in the plant. Moreover, the transition from the acidic stomach environment to the slightly alkaline intestinal environment resulted in a significant increase in the bioaccessibility of compounds, indicating that the intestinal conditions allowed the compounds to be released from the plant matrix and remain stable.^38^ It has been noted that most of the polyphenols found in herbal infusions were significantly reduced in the gastric phase and increased after intestinal phase, while some polyphenols were not detected after *in vitro* digestion. It is known that this situation is caused by the damage of some polyphenols since the pH and other environmental conditions of the gastric and intestinal phases may not be suitable.^22^

### 3.3 Changes in the Antioxidant Capacity during *In Vitro* Gastrointestinal Digestion

In Table 4, changes in the antioxidant capacity of herbal infusions during the simulation of *in vitro* digestion are shown. It was determined that there was a statistically significant variation in the total antioxidant capacity of herbal infusions (*p* < 0.05) for the undigested, *in vitro* gastric digested, and the *in vitro* intestinal digested samples.

**Table 4:** Effect of *In Vitro* Digestion Assay on Antioxidant Capacity of Herbal Infusions^a^

		Treatments			
Assays	Infusions	UD	GD	ID	% Bioaccessibility
DPPH (mg TE/100 g)	*R. canina* L.	621 ± 31 ^dC^	2465 ± 197^cB^	3892 ± 147 ^bA^	627 ± 24^a^
	*C. monogyna* Jacq.	539 ± 4 ^eC^	2375 ± 119 ^cdA^	2158 ± 109 ^cB^	400 ± 20^c^
	*C. orientalis* Pall.	316 ± 28 ^gB^	2104 ± 98 ^dA^	2126 ± 213 ^cA^	661 ± 75^a^
	*C. orientalis*	236 ± 18 ^hC^	1742 ± 80 ^eA^	1517 ± 160 ^dB^	642 ± 68^a^
	*I. persica* L.	680 ± 20 ^cC^	3932 ± 155^bA^	3585 ± 289 ^bB^	527 ± 42^b^
	*F. elaeochytris*	1236 ± 37 ^bC^	2702 ± 102^cA^	2127 ± 145 ^cB^	172 ± 12^e^
	*A. azurea* Mill.	2168 ± 44 ^aC^	9951 ± 393^aA^	8785 ± 574 ^aB^	405 ± 26^c^
	*E. spectabilis* M. Bieb.	514 ± 10 ^eC^	2656 ± 140^cA^	1704 ± 173 ^dB^	331 ± 34^d^
CUPRAC (mg TE/100 g)	*R. canina* L.	4333 ± 147 ^bB^	7375 ± 474 ^bA^	3565 ± 166 ^cC^	82 ± 4^ef^
	*C. monogyna* Jacq.	676 ± 102 ^dC^	3260 ± 375 ^deA^	1854 ± 108 ^efB^	274 ± 16^b^
	*C. orientalis* Pall.	187 ± 38 ^eC^	2128 ± 290 ^ef A^	1805 ± 86 ^efB^	968 ± 46^a^
	*C. orientalis*	735 ± 47 ^dB^	1648 ± 166 ^fA^	1738 ± 71 ^fA^	237 ± 10^c^
	*I. persica* L.	4361 ± 214 ^bB^	8321 ± 773 ^abA^	4639 ± 312^bB^ bB	102 ± 5^e^
	*F. elaeochytris*	4705 ± 203 ^bB^	5370 ± 568 ^cA^	2827 ± 237 ^dC^	76 ± 21^f^
	*A. azurea* Mill.	6441 ± 533 ^aB^	9059 ± 950a^A^	9059 ± 390 ^aA^	141 ± 6^d^
	*E. spectabilis* M. Bieb.	1523 ± 99 ^cC^	4436 ± 472 ^cdA^	2173 ± 101 ^eB^	143 ± 7^d^

a UD: undigested digestion, GD: gastric digestion, and ID: intestinal digestion; CUPRAC: cupric ion reducing antioxidant capacity; DPPH: 1,1-diphenyl-2-picrylhydrazyl; TE: Trolox equivalents. The data presented in this table consist of average values ± standard deviation of three independent batches. Different small letters indicate the differences between different herb species, while capital letters represent the statistically significant differences between the digestive phases of the same herb sample (*p* < 0.05).

*A. azurea* showed the highest antioxidant capacity values both by CUPRAC and DPPH assays (6441 ± 533 and 2168 ± 44 TE/100 g dw, respectively) compared to the other samples, which were well correlated with the total phenolic and total flavonoid contents. In a study, including *A. azurea* and *F. elaeochytris* samples, the antioxidant capacity of *A. azurea* was found to be higher than that of *F. elaeochytris*,^39^ which is similar to the data obtained in this study. The result of the antioxidant capacity of herbal infusions by the CUPRAC assay was significantly higher than the DPPH method. For instance, while the antioxidant capacity of *R. canina* obtained by CUPRAC assay was 4333 ± 147 TE/100 g dw, DPPH results showed that the antioxidant capacity of this sample was 621 ± 31TE/100 g dw. Similarly, Kilicgun and Altiner^24^ reported that a higher antioxidant capacity was observed for *R*. *canina* L. by the CUPRAC assay compared to the DPPH method.

Antioxidant capacity by the DPPH method increased in the following order: undigested < intestinal digestion < gastric digestion, whereas the order for the CUPRAC assay varied. The antioxidant potential may be different during the digestion condition because the radical scavenging capacity of phenolic compounds is based on the pH of the environment. Much of the radical scavenging capacity of phenolic compounds is dependent on the pH of the environment, which can cause fluctuations in antioxidant capacity during digestion.^22^ In addition, phenolic substances may undergo structural changes through the gastrointestinal passage due to the ionization of hydroxyl groups, which may lead to enhanced antioxidant capacity at higher pH values. Similarly, Tagliazucchi et al.^40^ found that the free radical scavenging activity of various phenolic compounds, such as gallic acid, caffeic acid, catechins, quercetin, and resveratrol, increased after intestinal digestion.

Ozkan et al.^41^ reported that there was a significant increase in the total antioxidant activity of different herbal infusions after *in vitro* gastric digestion, which is similar to the findings of the present study. It can be interpreted that an increase in the antioxidant capacity after gastric digestion implies some phenolic compounds revealed in the stomach have greater antioxidant activity. However, according to a study on the *in vitro* bioaccessibility of basil (*Ocimum basilicum* L.) phytochemicals conducted by Sęczyk et al.,^42^ the TPC values of different basil cultivars were decreased significantly during gastrointestinal digestion.

## 4 Conclusion

This study focused on the evaluation of the total phenolic and total flavonoid contents of the infusions of various herbs obtained from different regions of Turkey, indicating the stability and bioaccessibility of their phenolics during digestion by applying a standard static *in vitro* digestion protocol. Even though these plants are frequently consumed in these regions, the information presented in the literature is very limited. From the results obtained, the phenolic contents of the plant samples used in infusions were found to be comparatively high; especially *A. azurea* had the highest TPC (5836 ± 373 mg GAE/100 g dw), TFC (2301 ± 158 mg CE/100 g dw), and TAC (6441 ± 633 mg TE/100 g dw for the CUPRAC; 2168 ± 44 mg TE/100 g dw for the DPPH) values among others. In addition to these, gallic acid, chlorogenic acid, syringic acid, and rutin were the most frequently identified phenolic compounds in the samples. The highest bioaccessibility values were determined in *C. orientalis* and *C. orientalis* Pall samples. The results of this study showed that these plants can be an alternative to the most widely consumed herbal teas, with their high and stable phenolic and flavonoid contents. In future studies, using Caco-2 cell culture models together with *in vitro* gastrointestinal digestion method will provide more information on the fate of phenolics in these plant infusions.

## Supporting Information Available

Chromatograms for all plants used in this study (PDF)

## References

[ref1] Martins N.; Barros L.; Ferreira I. C. F. R. In Vivo Antioxidant Activity of Phenolic Compounds: Facts and Gaps. Trends in Food Science & Technology 2016, 48, 1–12. 10.1016/j.tifs.2015.11.008.

[ref2] Murathan Z.; Arslan M.; Erbil N. EVALUATION OF ANTIOXIDANT, ANTIMICROBIAL AND ANTIMUTAGENIC PROPERTIES IN Eremurus Spectabilis BIEB. ARE GROWN IN DIFFERENT ECOLOGICAL REGIONS. Fresenius Environ. Bull. 2018, 27, 9491.

[ref3] Hinneburg I.; Damien Dorman H. J.; Hiltunen R. Antioxidant Activities of Extracts from Selected Culinary Herbs and Spices. Food Chem. 2006, 97 (1), 122–129. 10.1016/j.foodchem.2005.03.028.

[ref4] Winiarska-Mieczan A.; Kwiecień M.; Jachimowicz-Rogowska K.; Donaldson J.; Tomaszewska E.; Baranowska-Wójcik E. Anti-Inflammatory, Antioxidant, and Neuroprotective Effects of Polyphenols—Polyphenols as an Element of Diet Therapy in Depressive Disorders. International Journal of Molecular Sciences 2023, 24 (3), 2258 10.3390/ijms24032258. 36768580 PMC9916817

[ref5] Li X.; Huang W.; Tan R.; Xu C.; Chen X.; Li S.; Liu Y.; Qiu H.; Cao H.; Cheng Q. The Benefits of Hesperidin in Central Nervous System Disorders, Based on the Neuroprotective Effect. Biomedicine & Pharmacotherapy 2023, 159, 114222 10.1016/j.biopha.2023.114222. 36628819

[ref6] Ozkan G.; Kamiloglu S.; Ozdal T.; Boyacioglu D.; Capanoglu E. Potential Use of Turkish Medicinal Plants in the Treatment of Various Diseases. Molecules 2016, 21 (3), 257 10.3390/molecules21030257. 26927038 PMC6273156

[ref7] Ozkan G.; Stübler A.-S.; Aganovic K.; Dräger G.; Esatbeyoglu T.; Capanoglu E. Retention of Polyphenols and Vitamin C in Cranberrybush Purée (Viburnum Opulus) by Means of Non-Thermal Treatments. Food Chem. 2021, 360, 129918 10.1016/j.foodchem.2021.129918. 34051454

[ref8] Ramesh A.; Varghese S. S.; Doraiswamy J. N.; Malaiappan S. Herbs as an Antioxidant Arsenal for Periodontal Diseases. J. Intercult Ethnopharmacol 2016, 5 (1), 92–96. 10.5455/jice.20160122065556. 27069730 PMC4805154

[ref9] Kamiloglu S.; Capanoglu E.; Yilmaz O.; Duran A. f.; Boyacioglu D. Investigating the Antioxidant Potential of Turkish Herbs and Spices. Quality Assurance and Safety of Crops & Foods 2014, 6 (2), 151–158. 10.3920/QAS2012.0237.

[ref10] Leahu A.; Damian C.; Oroian M.; Ropciuc S.; Rotaru R. Influence of Processing on Vitamin C Content of Rosehip Fruits. Scientific Papers: Animal Science and Biotechnologies 2014, 47 (1), 47.

[ref11] Suna S. Investigating the Physicochemical Properties and in Vitro Bioaccessibility of Phenolics and Antioxidant Capacity of Rooibos Herbal Tea Beverage. Gida/the Journal of Food 2017, 42 (6), 682–692. 10.15237/gida.GD17050.

[ref12] Celep E.; Akyuz S.; İnan Y.; Yesilada E. Stability of Phenolic Content of Some Herbal Infusions and Their Antioxidant Activity Following in Vitro Digestion. Turkish Journal of Biochemistry 2017, 42 (4), 375–380. 10.1515/tjb-2017-0178.

[ref13] İlyasoğlu H.; Arpa T. E. Effect of Brewing Conditions on Antioxidant Properties of Rosehip Tea Beverage: Study by Response Surface Methodology. J. Food Sci. Technol. 2017, 54 (11), 3737–3743. 10.1007/s13197-017-2794-2. 29051670 PMC5629151

[ref14] Capanoglu E.; Beekwilder J.; Boyacioglu D.; Hall R.; de Vos R. Changes in Antioxidant and Metabolite Profiles during Production of Tomato Paste. J. Agric. Food Chem. 2008, 56 (3), 964–973. 10.1021/jf072990e. 18205308

[ref15] Minekus M.; Alminger M.; Alvito P.; Ballance S.; Bohn T.; Bourlieu C.; Carrière F.; Boutrou R.; Corredig M.; Dupont D.; Dufour C.; Egger L.; Golding M.; Karakaya S.; Kirkhus B.; Feunteun S. L.; Lesmes U.; Macierzanka A.; Mackie A.; Marze S.; McClements D. J.; Ménard O.; Recio I.; Santos C. N.; Singh R. P.; Vegarud G. E.; Wickham M. S. J.; Weitschies W.; Brodkorb A. A Standardised Static in Vitro Digestion Method Suitable for Food – an International Consensus. Food Funct. 2014, 5 (6), 1113–1124. 10.1039/C3FO60702J. 24803111

[ref16] Singleton V. L.; Rossi J. A. Colorimetry of Total Phenolics with Phosphomolybdic-Phosphotungstic Acid Reagents. Am. J. Enol. Vitic. 1965, 16 (3), 144–158.

[ref17] Dewanto V.; Wu X.; Adom K. K.; Liu R. H. Thermal Processing Enhances the Nutritional Value of Tomatoes by Increasing Total Antioxidant Activity. J. Agric. Food Chem. 2002, 50 (10), 3010–3014. 10.1021/jf0115589. 11982434

[ref18] Brand-Williams W.; Cuvelier M. E.; Berset C. Use of a Free Radical Method to Evaluate Antioxidant Activity. LWT - Food Science and Technology 1995, 28 (1), 25–30. 10.1016/S0023-6438(95)80008-5.

[ref19] Khomsi M. E.; Imtara H.; Kara M.; Hmamou A.; Assouguem A.; Bourkhiss B.; Tarayrah M.; AlZain M. N.; Alzamel N. M.; Noman O.; Hmouni D. Antimicrobial and Antioxidant Properties of Total Polyphenols of Anchusa Italica Retz. Molecules 2022, 27 (2), 416 10.3390/molecules27020416. 35056731 PMC8778933

[ref20] Murathan Z. T.; Özdi̇nç M. Ardahan ve Elazığ illerinde Yetişen Anchusa azurea Miller var. Azurea Bitkisinin Biyoaktif Bileşenleri ve Antioksidan Kapasitesi Üzerine Bir Araştırma. Kahramanmaraş Sütçü İmam Üniversitesi Tarım ve Doğa Dergisi 2018, 21 (4), 529–534. 10.18016/ksudobil.362296.

[ref21] Javi̇d D.; Ak-Sakalli̇ E.; Ozti̇nen N.; Atli̇ B.; Kosar M. ANTIRADICAL ACTIVITIES AND PHENOLIC COMPOSITIONS OF ROSA CANINA L. FROM IRAN AND TURKEY. EMU Journal of Pharmaceutical Sciences 2021, 4 (1), 28–35.

[ref22] Ozkan G.; Franco P.; De Marco I.; Capanoglu E.; Esatbeyoglu T. Investigating the Effects of Supercritical Antisolvent Process and Food Models on Antioxidant Capacity, Bioaccessibility and Transepithelial Transport of Quercetin and Rutin. Food and Function 2022, 13, 4469 10.1039/D1FO04091J. 35343983

[ref23] Petkova N.; Ivanova L.; Filova G.; Ivanov I.; Denev P. Antioxidants and Carbohydrate Content in Infusions and Microwave Extracts from Eight Medicinal Plants. Journal of Applied Pharmaceutical Science 2017, 7 (10), 55–61. 10.7324/JAPS.2017.71008.

[ref24] Kilicgun H.; Altiner D. Correlation between Antioxidant Effect Mechanisms and Polyphenol Content of Rosa Canina. Pharmacognosy Magazine 2010, 6 (23), 238 10.4103/0973-1296.66943. 20931086 PMC2950389

[ref25] Przybylska A.; Bazylak G. Bioactive Compounds in Aqueous Infusions of Dietary Supplements and Herbal Blends Containing Dried Hawthorn Fruits or Hawthorn Inflorescences (Crataegus spp.). JAES 2018, 7 (2), n2a14 10.15640/jaes.v7n2a14.

[ref26] Qin Y.; Wang L.; Liu Y.; Zhang Q.; Li Y.; Wu Z. Release of Phenolics Compounds from Rubus Idaeus L. Dried Fruits and Seeds during Simulated in Vitro Digestion and Their Bio-Activities. Journal of Functional Foods 2018, 46, 57–65. 10.1016/j.jff.2018.04.046.

[ref27] Lou X.; Guo X.; Wang K.; Wu C.; Jin Y.; Lin Y.; Xu H.; Hanna M.; Yuan L. Phenolic Profiles and Antioxidant Activity of Crataegus Pinnatifida Fruit Infusion and Decoction and Influence of in Vitro Gastrointestinal Digestion on Their Digestive Recovery. LWT 2021, 135, 110171 10.1016/j.lwt.2020.110171.

[ref28] Seiquer I.; Rueda A.; Olalla M.; Cabrera-Vique C. Assessing the Bioavailability of Polyphenols and Antioxidant Properties of Extra Virgin Argan Oil by Simulated Digestion and Caco-2 Cell Assays. Comparative Study with Extra Virgin Olive Oil. Food Chem. 2015, 188, 496–503. 10.1016/j.foodchem.2015.05.006. 26041223

[ref29] Campos-Vega R.; Vázquez-Sánchez K.; López-Barrera D.; Loarca-Piña G.; Mendoza-Díaz S.; Oomah B. D. Simulated Gastrointestinal Digestion and in Vitro Colonic Fermentation of Spent Coffee (Coffea Arabica L.): Bioaccessibility and Intestinal Permeability. Food Research International 2015, 77, 156–161. 10.1016/j.foodres.2015.07.024.

[ref30] Ozkan G.; Kostka T.; Dräger G.; Capanoglu E.; Esatbeyoglu T. Bioaccessibility and Transepithelial Transportation of Cranberrybush (Viburnum Opulus) Phenolics: Effects of Non-Thermal Processing and Food Matrix. Food Chem. 2022, 380 (September 2021), 132036 10.1016/j.foodchem.2021.132036. 35101787

[ref31] Polumackanycz M.; Kaszuba M.; Konopacka A.; Marzec-Wróblewska U.; Wesolowski M.; Waleron K.; Buciński A.; Viapiana A. Phenolic Composition and Biological Properties of Wild and Commercial Dog Rose Fruits and Leaves. Molecules 2020, 25 (22), 5272 10.3390/molecules25225272. 33198171 PMC7697969

[ref32] Alirezalu A.; Ahmadi N.; Salehi P.; Sonboli A.; Alirezalu K.; Khaneghah A. M.; Barba F. J.; Munekata P. E. S.; Lorenzo J. M. Physicochemical Characterization, Antioxidant Activity, and Phenolic Compounds of Hawthorn (Crataegus spp.) Fruits Species for Potential Use in Food Applications. Foods 2020, 9 (4), 436 10.3390/foods9040436. 32260449 PMC7230283

[ref33] Ozkan G.; Esatbeyoglu T.; Capanoglu E. Bioavailability of Rosehip (Rosa Canina L.) Infusion Phenolics Prepared by Thermal, Pulsed Electric Field and High-Pressure Processing. Foods 2022, 11 (13), 1955 10.3390/foods11131955. 35804770 PMC9265957

[ref34] Ben Hlel T.; Borges T.; Rueda A.; Smaali I.; Marzouki M. N.; Seiquer I. Polyphenols Bioaccessibility and Bioavailability Assessment in Ipecac Infusion Using a Combined Assay of Simulated in Vitro Digestion and Caco-2 Cell Model. International Journal of Food Science & Technology 2019, 54 (5), 1566–1575. 10.1111/ijfs.14023.

[ref35] Capanoglu E.; Kamiloglu S.; Ozkan G.; Apak R. Evaluation of Antioxidant Activity/Capacity Measurement Methods for Food Products. In Measurement of Antioxidant Activity & Capacity; John Wiley & Sons, Ltd., 2018; pp 273–286. 10.1002/9781119135388.ch13.

[ref36] Ho Y.-C.; Yu H.-T.; Su N.-W. Re-Examination of Chromogenic Quantitative Assays for Determining Flavonoid Content. J. Agric. Food Chem. 2012, 60 (10), 2674–2681. 10.1021/jf2045153. 22352692

[ref37] Zheng G.; Deng J.; Wen L.; You L.; Zhao Z.; Zhou L. Release of Phenolic Compounds and Antioxidant Capacity of Chinese Hawthorn “Crataegus Pinnatifida” during in Vitro Digestion. Journal of Functional Foods 2018, 40, 76–85. 10.1016/j.jff.2017.10.039.

[ref38] de Paulo Farias D.; de Araújo F. F.; Neri-Numa I. A.; Dias-Audibert F. L.; Delafiori J.; Catharino R. R.; Pastore G. M. Effect of in Vitro Digestion on the Bioaccessibility and Bioactivity of Phenolic Compounds in Fractions of Eugenia Pyriformis Fruit. Food Research International 2021, 150, 110767 10.1016/j.foodres.2021.110767. 34865782

[ref39] Samancioglu A.; Sat I. G.; Yildirim E.; Jurikova T.; Mlcek J. Total Phenolic and Vitamin C Content and Antiradical Activity Evaluation of Traditionally Consumed Wild Edible Vegetables from Turkey. Indian J. Trad. Knowledge 2016, 15, 208–213.

[ref40] Tagliazucchi D.; Verzelloni E.; Bertolini D.; Conte A. In Vitro Bio-Accessibility and Antioxidant Activity of Grape Polyphenols. Food Chem. 2010, 120 (2), 599–606. 10.1016/j.foodchem.2009.10.030.

[ref41] Özkan G.; Aras A.; Güven E. Ç. Investigating the Antioxidant Properties of Some Herbal Infusions During In Vitro Digestion. Journal of Apitherapy and Nature 2022, 5 (1), 1–13. 10.35206/jan.1106268.

[ref42] Sęczyk Ł.; Ozdemir F. A.; Kołodziej B. In Vitro Bioaccessibility and Activity of Basil (Ocimum Basilicum L.) Phytochemicals as Affected by Cultivar and Postharvest Preservation Method – Convection Drying, Freezing, and Freeze-Drying. Food Chem. 2022, 382, 132363 10.1016/j.foodchem.2022.132363. 35158270

