# Supplementary material for: Effect of In Vitro Digestion on the Phenolic Content of Herbs Collected from Eastern Anatolia
Source: ACS Omega. 2023 Mar 28;8(14):12730–8. doi: 10.1021/acsomega.2c07881 (PMC10099410; doi:10.1021/acsomega.2c07881)
Supplement: Supplementary file 1 — ao2c07881_si_001.pdf [file ao2c07881_si_001.pdf]

Effect of *In Vitro* Digestion on the Phenolic Content of Herbs Collected from Eastern Anatolia

Gulay Ozkan[1], Fatma Betul Sakarya[1], Dilara Tas[1], Bayram Yurt[2], Sezai Ercisli[3], Esra Capanoglu[1]*

[1]Department of Food Engineering, Faculty of Chemical and Metallurgical Engineering, Istanbul Technical University, 34469 Maslak, Istanbul, Turkey

[2]Department of Food Engineering, Faculty of Engineering and Architecture, Bingöl University, Bingöl, Turkey

[3]Department of Horticulture, Faculty of Agriculture, Ataturk University 25240, Erzurum, Turkey

* Corresponding author: Esra Capanoglu, capanogl@itu.edu.tr

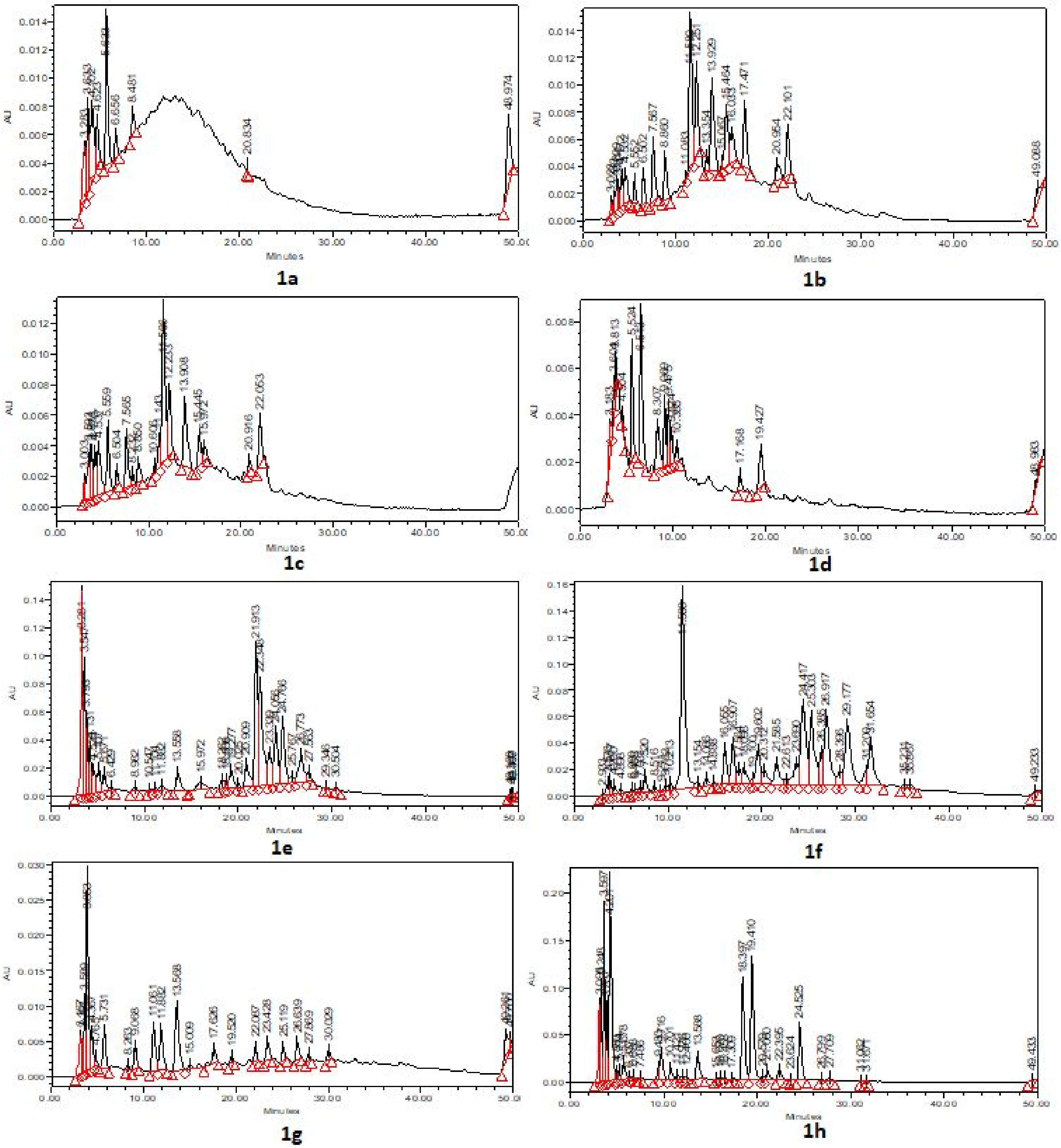

Figure S1. Chromatogram for (a) *R. canina L.,* (b) *C. monogyna Jacq.*, (c) *C. orientalis Pall.*, (d) *C. orientalis*, (e) *I. persica L.*, (f) *F. elaeochytris*, (g) *A. azurea Mill.*, (h) *E. spectabilis M. Bieb.* at 280 nm
